# Supplementary material for: Cytomegalovirus Genetic Diversity and Evolution: Insights into Genotypes and Their Role in Viral Pathogenesis
Source: Pathogens. 2025 Jan 9;14(1):50. doi: 10.3390/pathogens14010050 (PMC11768282; doi:10.3390/pathogens14010050)
Supplement: Supplementary file 1 [file pathogens-14-00050-s001.zip › pathogens-3221773-supplementary.pdf]

Supplementary material

UL55

Supp Figure S1A. All possible alleles' combinations (haplotypes) of regions 22, 23 and 24 in UL55. Three alleles were identified in each region. Twelve haplotypes were identified.

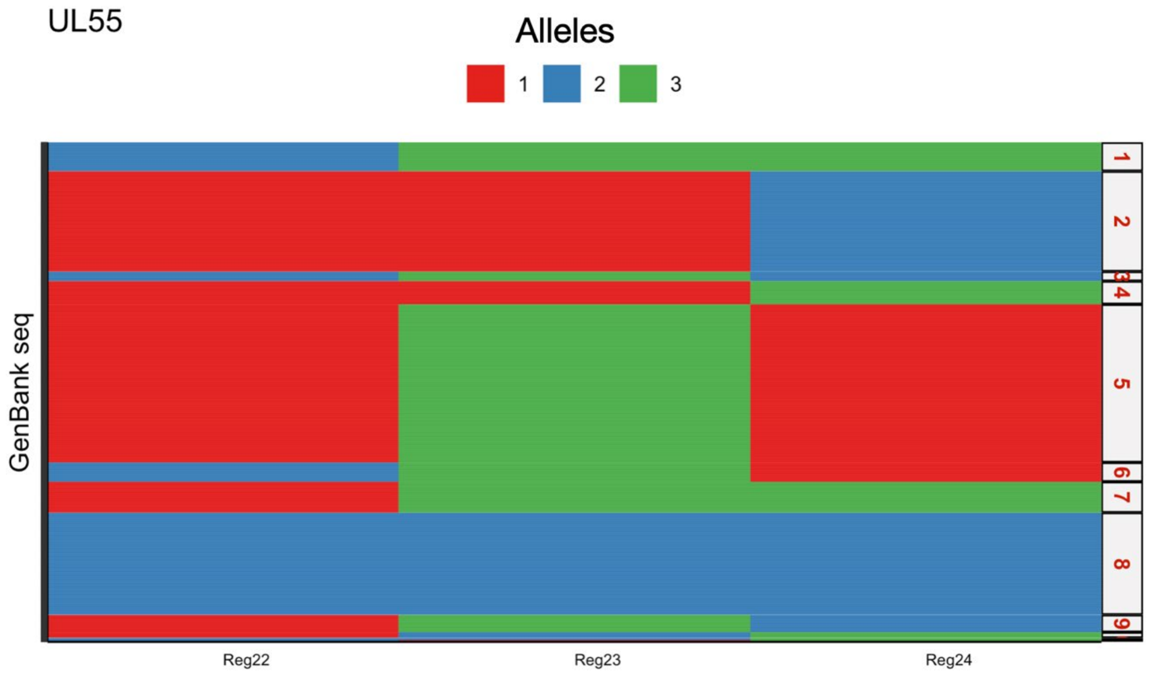

Supp Figure S1B. Frequency of haplotypes in UL55 in 253 GenBank sequences. Haplotypes 11 and 12 were only found in three African sequences (each) reconstructed from mixed infection with HaROLD (Charles et al., 2023; Venturini et al., 2022).

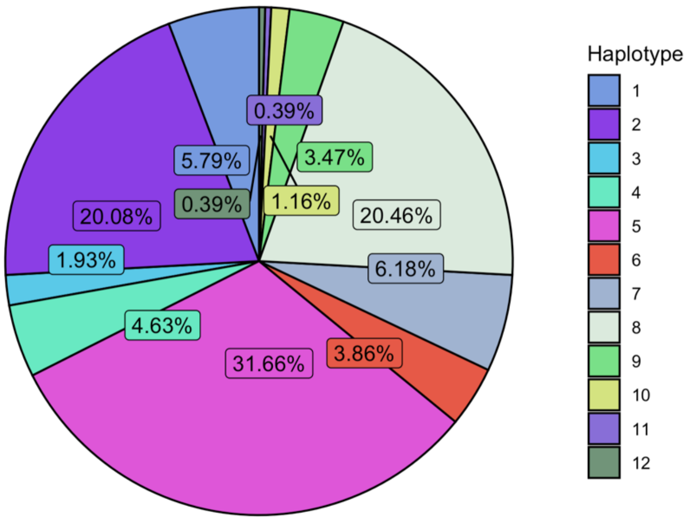

Supp Figure S1C. Frequency of haplotypes by continent in UL55 in 253 GenBank sequences.

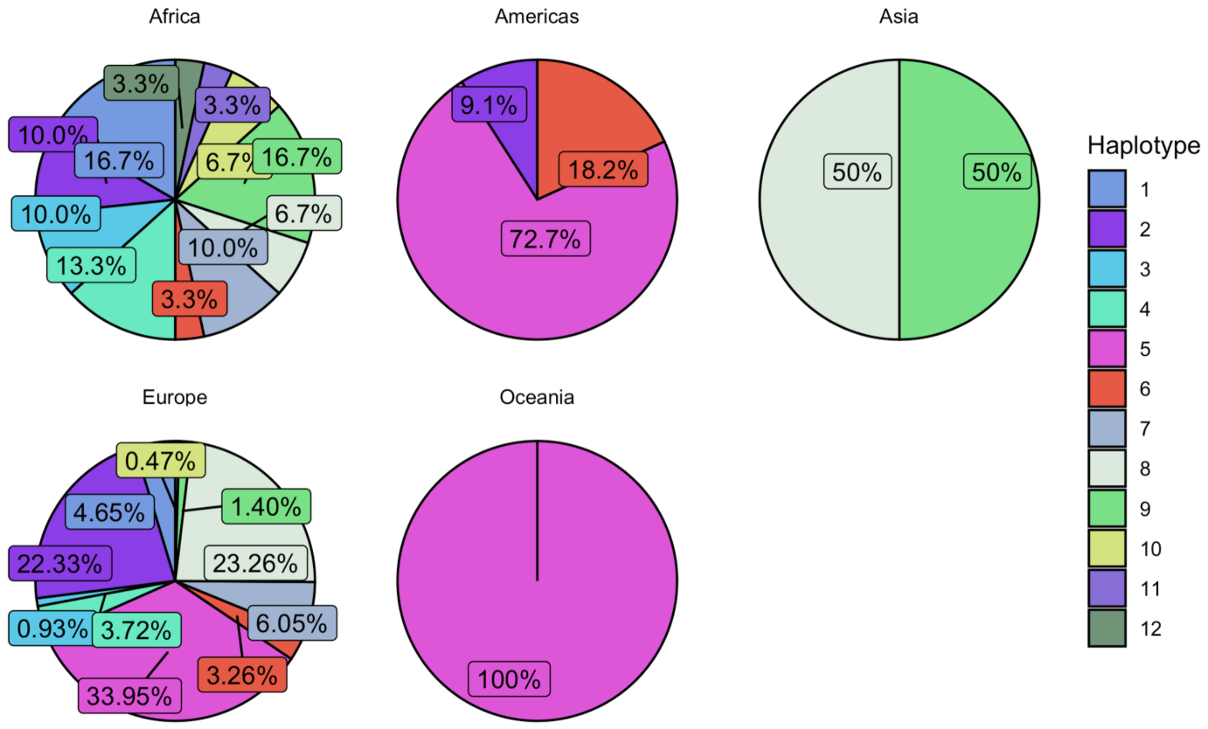

Supp Figure S1D. Phylogenetic tree (Neighbour joining) of representative sequences for the haplotypes (H1-H10) and genotypes (gB-1- gB-5).

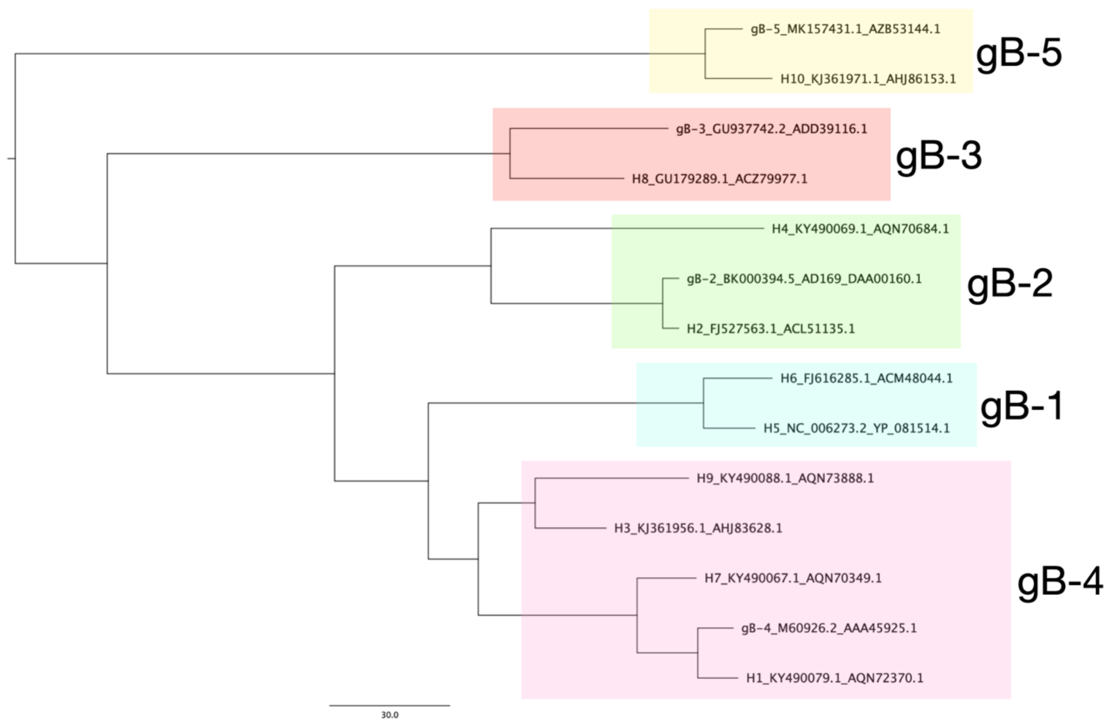

UL73 and UL74

Supp Figure S2A. Frequency of alleles in regions 28 comprising UL73/UL74 in 253 GenBank sequences

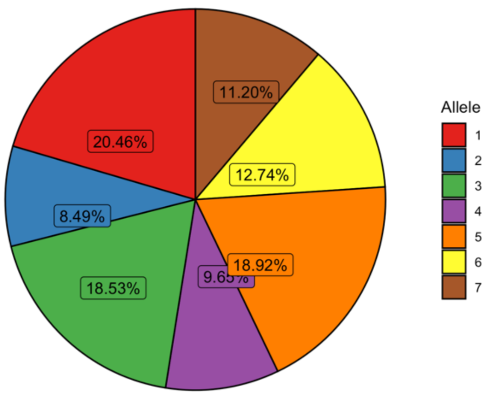

Supp Figure S2B. Frequency of alleles in regions 28 comprising UL73/UL74 in 253 GenBank sequences

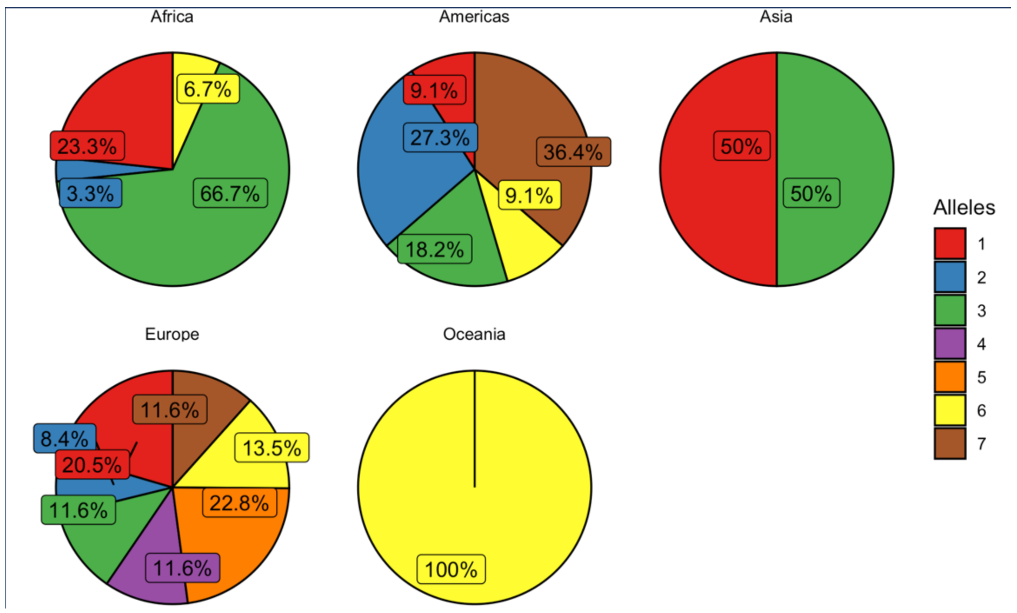

Supp Figure S2C. *Phylogenetic tree (Neighbour joining) of representative sequences for the alleles in region 28 (A1-A7) and gN genotypes (gN-1- gN-4 + subtypes).*

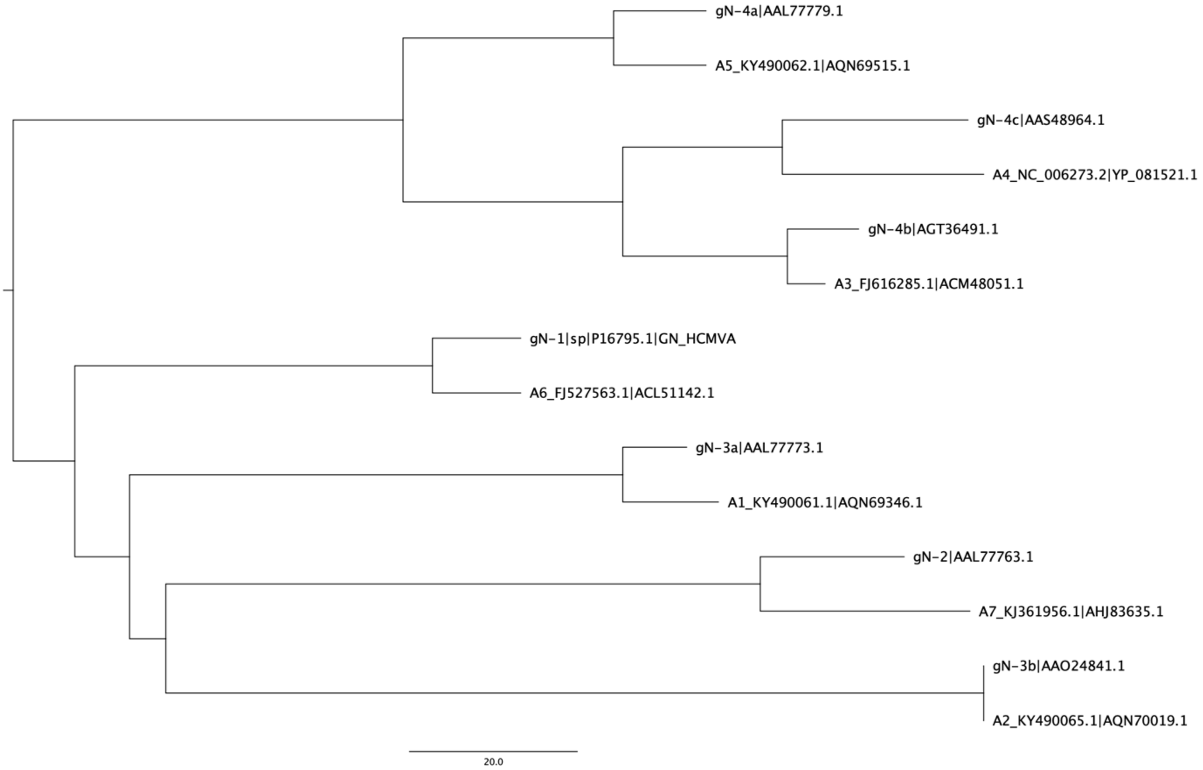

Supp Figure S3. Phylogenetic tree (Neighbour joining) of representative sequences for the alleles in region 28 (A1-A7) and gO genotypes.

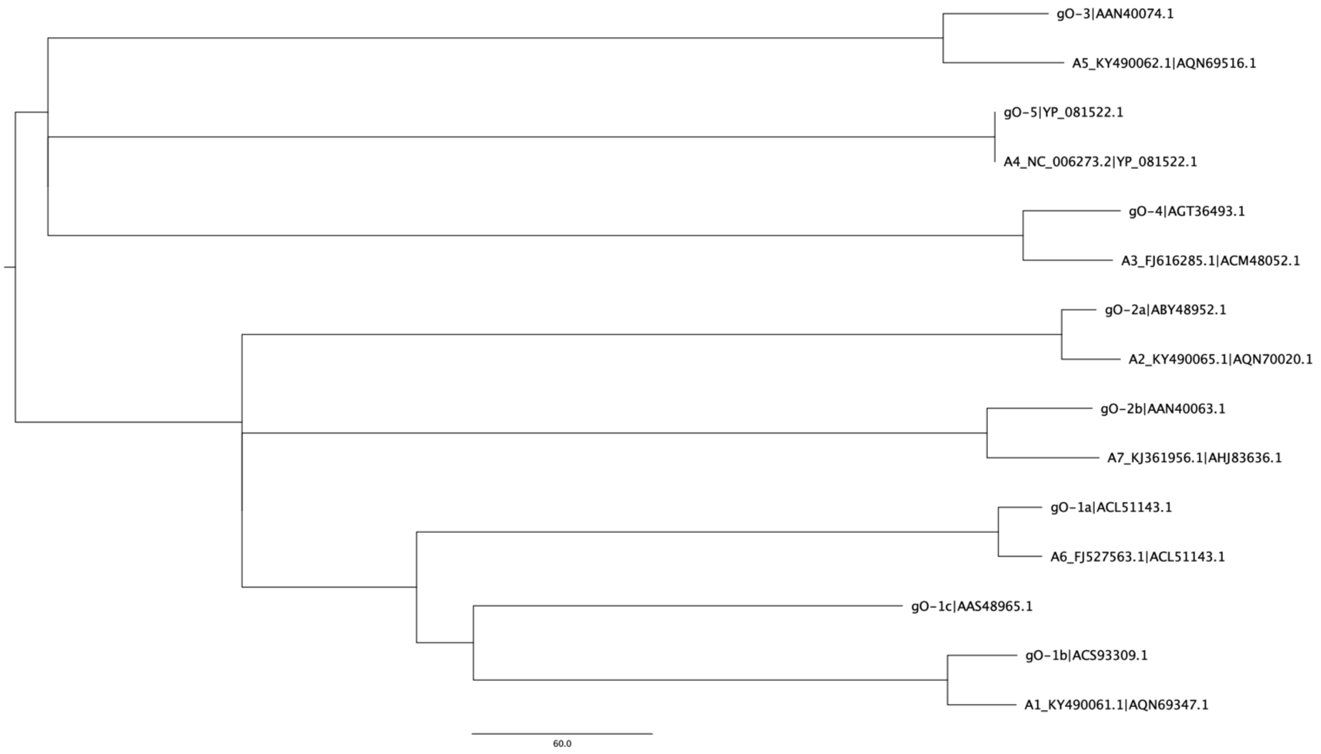

UL75

Supp Figure S4A. All possible alleles' combinations (haplotypes) of regions 29, 30 and 31 in UL75. Three alleles were identified in each region. Seven haplotypes were identified.

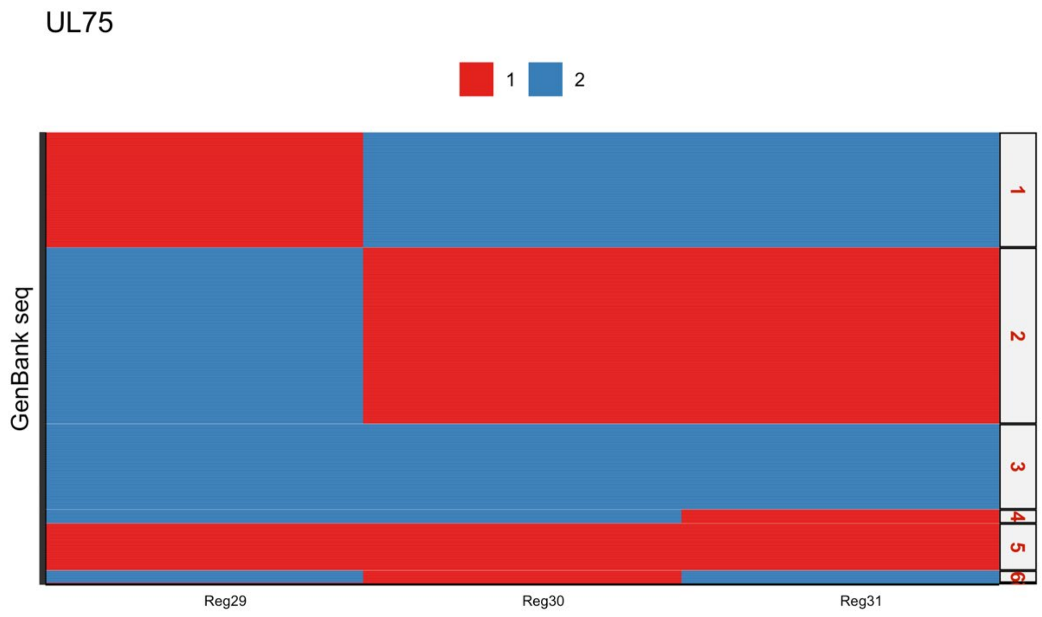

Supplementary Figure S4B. Frequency of haplotypes of regions 29, 30 and 31 in UL75 by continent in 253 GenBank sequences.

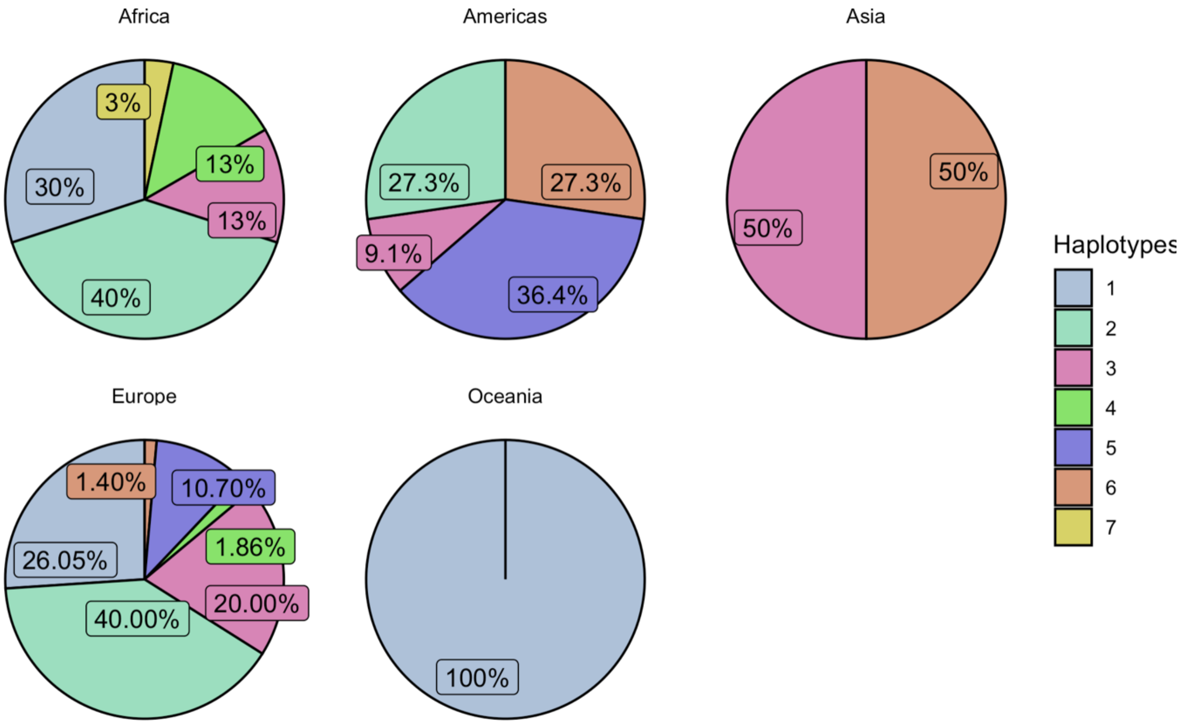

Supplementary Figure S4C. Phylogenetic tree (Neighbour joining) of representative sequences for the haplotypes in regions 29,30 and 31 and gH genotypes.

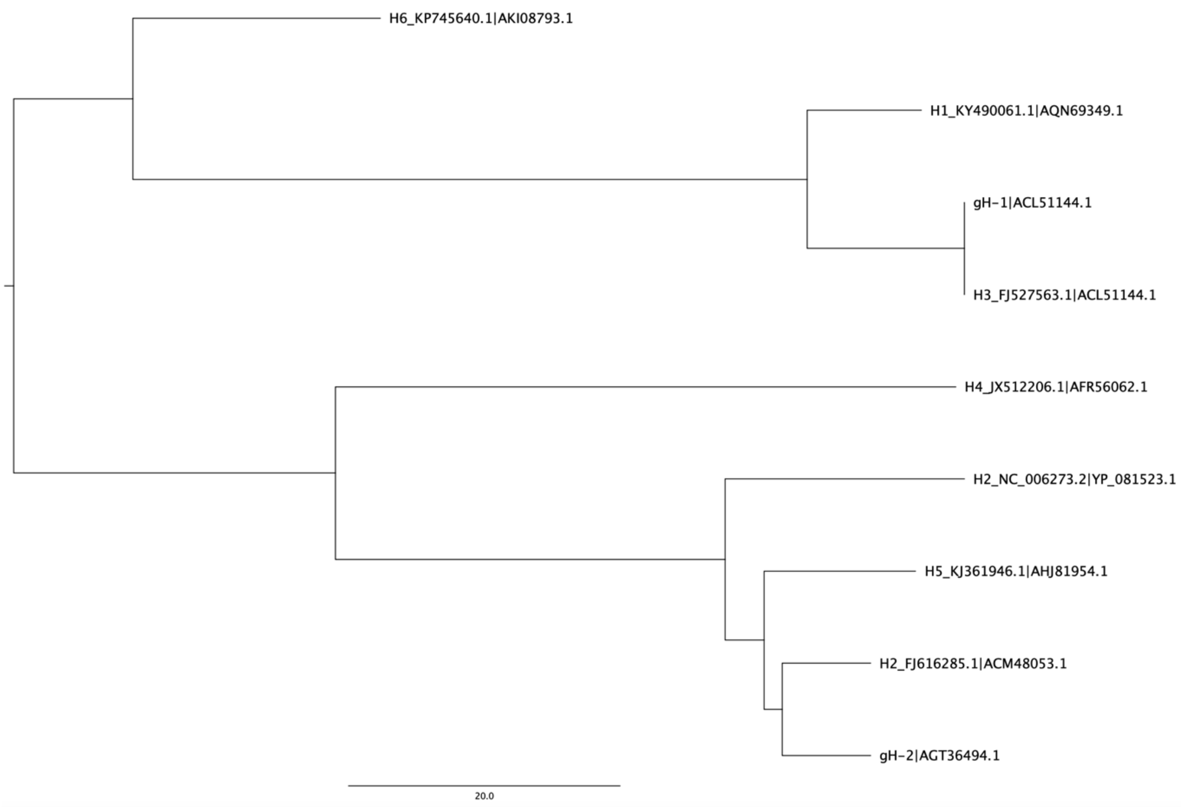

UL144

Supp Figure S5A. Frequency of alleles in region 50 comprising UL144 in 253 GenBank sequences

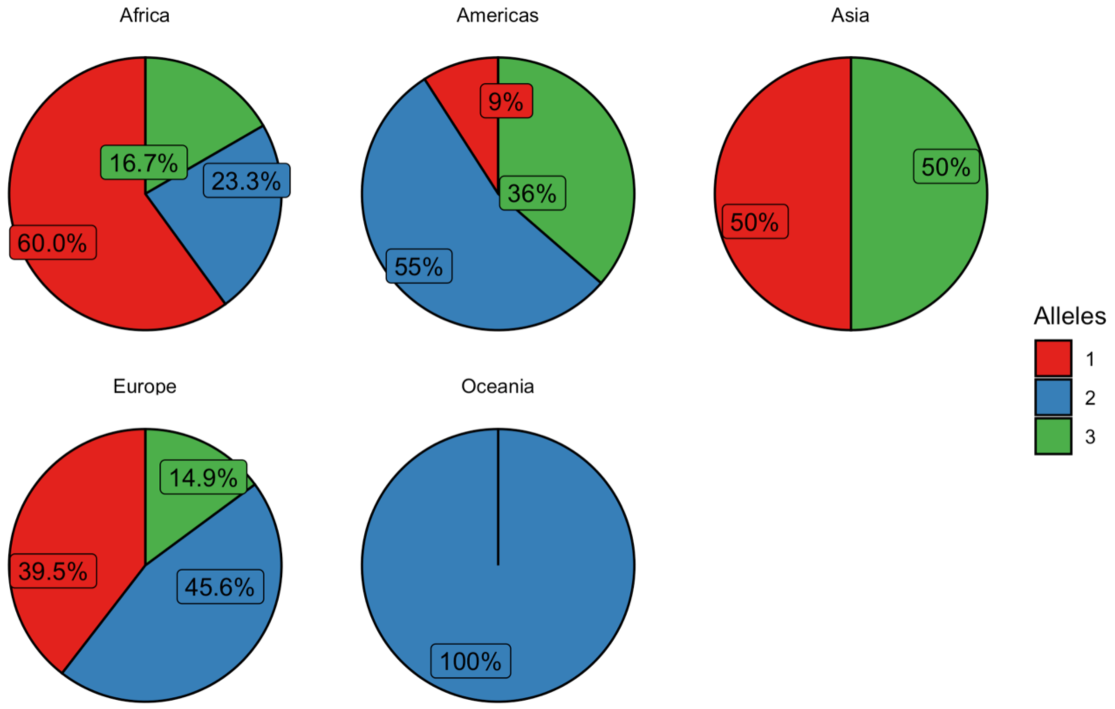

Supplementary Figure S5B. Phylogenetic tree (Neighbour joining) of representative sequences for UL144 alleles and genotypes.

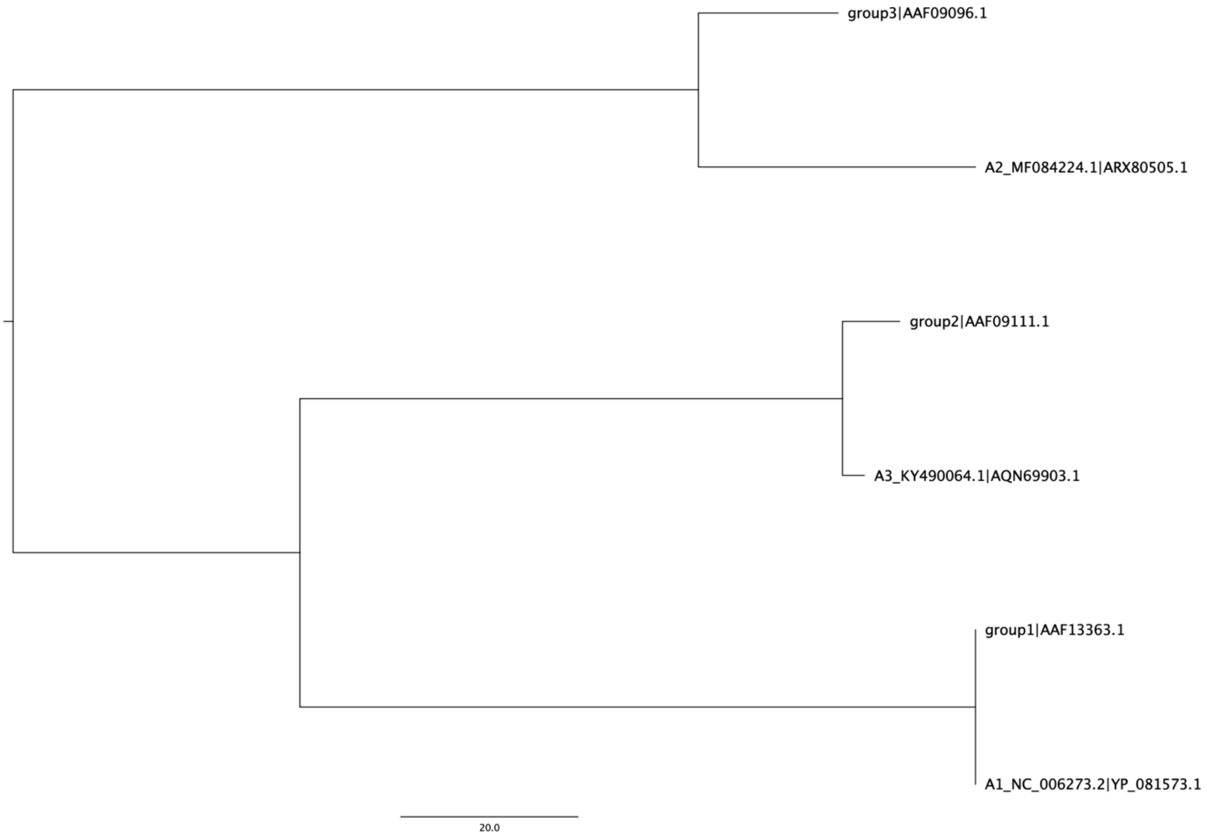

UL146

Supplementary Figure S6A. Frequency of alleles in UL146 by continent in 253 GenBank sequences.

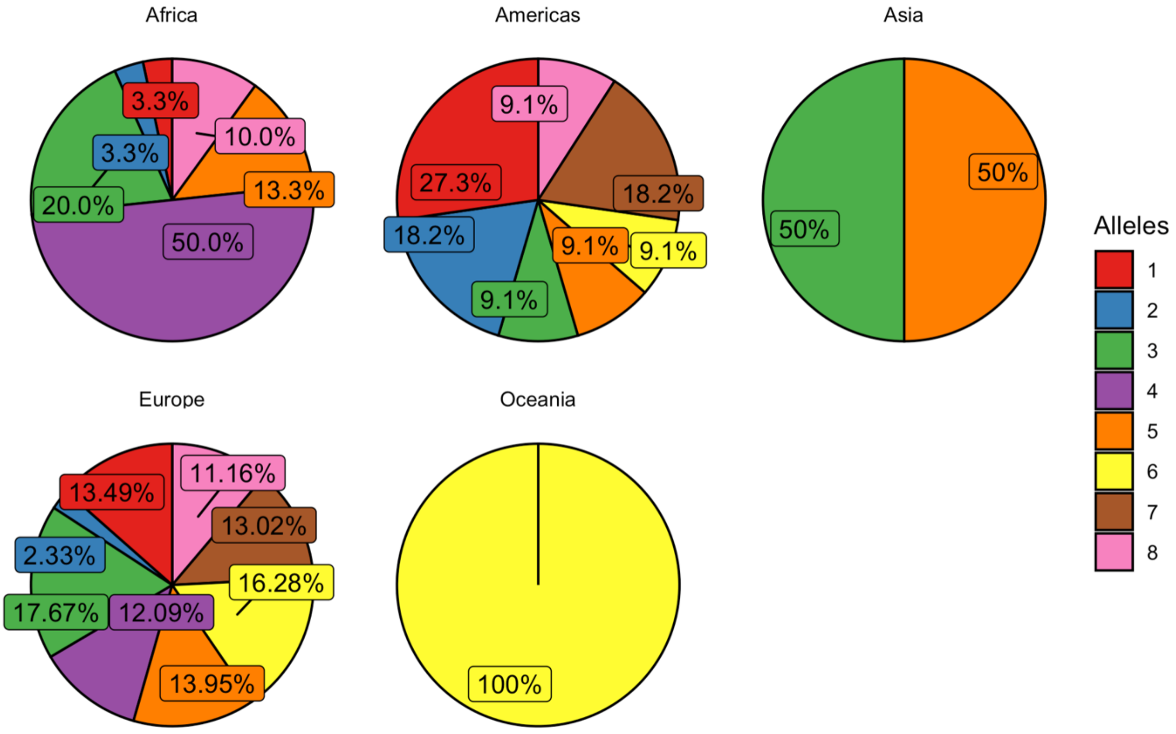

Supplementary Figure S6B. Phylogenetic tree (Neighbour joining) of representative sequences for UL146-UL147 alleles and genotypes.

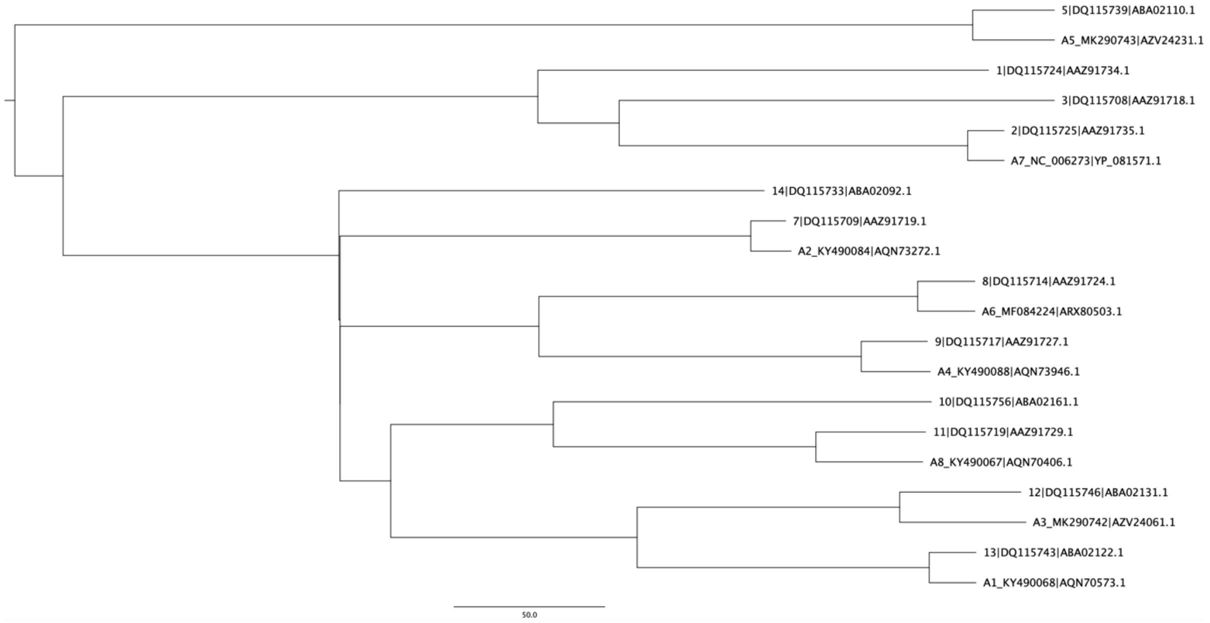

Supplementary Table S1. Novel multi-allelic regions and genes identified by Charles, Venturini et al. (Charles et al., 2023). The table shows novel multi-allelic regions with information about the open reading frame (gene) overlapping with the region, the protein encoded, the number of alleles and the geographic distribution between European and African sequences.

Region	Genes	Protein	Alleles n	Geography
9	UL18	membrane glycoprotein UL18	2	
11	UL22A	glycoprotein UL22A	3	
12	UL25	tegument protein UL25	2	
13	UL27	NA/ low-level resistance to maribavir	2	Yes
14	UL33	envelope glycoprotein UL33	3	
15	UL36	tegument protein vICA	2	
16	UL37	envelope glycoprotein UL37	5	Yes
18	UL41A/UL42	NA/ contains potential transmembrane domain	2	
19	UL45	ribonucleotide reductase subunit 1/ enzymatically inactive; tegument protein	2	Yes
20	UL48	large tegument protein	2	Yes
21	UL48A	small capsid protein	2	Yes
25			2	Yes
26		OriLyt-associated repeat/ Regulatory TATA box	2	Yes
27	RNA4.9	ncRNA	2	Yes
32	UL76	nuclear protein UL24	2	Yes
33	UL77	DNA packaging tegument protein UL25	2	Yes
34	UL78	envelope protein UL78	2	Yes
35	UL80/UL80.5	capsid maturation protease/ capsid scaffold protein	2	Yes
36	UL82	tegument protein pp71	2	Yes
37	UL86	major capsid protein	2	Yes
38	UL86		2	Yes
39	UL86		2	Yes
40	UL86		2	Yes
41	UL100	envelope glycoprotein M	2	Yes
42	UL116	NA/ contains signal peptide	2	
44	UL122		2	

45	UL122 UL123 UL124	regulatory protein IE2/IE1 and membrane protein UL124	2	
46	UL122 UL123 UL124		2	Yes
47	UL124		2	
48	UL132 UL148	envelope glycoprotein UL132/membrane protein UL148	2	
51	UL150A	regulatory TATA box	2	
52	UL142	membrane glycoprotein UL142	3	
54	UL133	NA/contains potential transmembrane domain	4	Yes
55	UL148A UL150A	NA contains potential transmembrane domain/NA polyA_signal_sequence	2	Yes
56	UL150	NA/contains signal peptide	2	
57	UL150		3	Yes
58	UL150		2	Yes
59	UL150		2	Yes
60	IRS1	tegument protein IRS1	2	
61	IRS1		2	
62	US7	membrane glycoprotein US7	2	
63	US7		2	
64	US14	membrane protein US14	2	
65	US17	membrane protein US17	2	Yes
70	US30	membrane protein US30	2	
71	US33A US34	TATA box NA/contains signal peptide	2	
72	TRS1	tegument protein TRS1	4	
73	TRS1		2	Yes
74	TRS1		2	

References:

Charles, O. J., Venturini, C., Gantt, S., Atkinson, C., Griffiths, P., Goldstein, R. A., & Breuer, J. (2023). Genomic and geographical structure of human cytomegalovirus. *Proceedings of the National Academy of Sciences*, *120*(30), e2221797120. https://doi.org/10.1073/pnas.2221797120

Venturini, C., Pang, J., Tamuri, A. U., Roy, S., Atkinson, C., Griffiths, P., Breuer, J., & Goldstein, R. A. (2022). Haplotype assignment of longitudinal viral deep-sequencing data using co-variation of variant frequencies. *Virus Evolution*. https://doi.org/10.1093/ve/veac093
